# An Underwater Positioning System for UUVs Based on LiDAR Camera and Inertial Measurement Unit

**DOI:** 10.3390/s22145418

**Published:** 2022-07-20

**Authors:** Hongbo Yang, Zhizun Xu, Baozhu Jia

**Affiliations:** Maritime College, Guangdong Ocean University, Zhanjiang 524000, China; 2112010001@stu.gdou.edu.cn (H.Y.); zhizun@gdou.edu.cn (Z.X.)

**Keywords:** underwater positioning, visual-inertial odometry, information fusion, ultra-wideband positioning system

## Abstract

Underwater positioning presents a challenging issue, because of the rapid attenuation of electronic magnetic waves, the disturbances and uncertainties in the environment. Conventional methods usually employed acoustic devices to localize Unmanned Underwater Vehicles (UUVs), which suffer from a slow refresh rate, low resolution, and are susceptible to the environmental noise. In addition, the complex terrain can also degrade the accuracy of the acoustic navigation systems. The applications of underwater positioning methods based on visual sensors are prevented by difficulties of acquiring the depth maps due to the sparse features, the changing illumination condition, and the scattering phenomenon. In the paper, a novel visual-based underwater positioning system is proposed based on a Light Detection and Ranging (LiDAR) camera and an inertial measurement unit. The LiDAR camera, benefiting from the laser scanning techniques, could simultaneously generate the associated depth maps. The inertial sensor would offer information about its altitudes. Through the fusion of the data from multiple sensors, the positions of the UUVs can be predicted. After that, the Bundle Adjustment (BA) method is used to recalculate the rotation matrix and the translation vector to improve the accuracy. The experiments are carried out in a tank to illustrate the effects and accuracy of the investigated method, in which the ultra-wideband (UWB) positioning system is used to provide reference trajectories. It is concluded that the developed positioning system is able to estimate the trajectory of UUVs accurately, whilst being stable and robust.

## 1. Introduction

Underwater positioning systems are crucial to Autonomous Underwater Vehicles (AUVs), Remote Operated Vehicles (ROVs) and other Unmanned Underwater Vehicles (UUVs). The underwater robotics are well-known for being applied in the deep-water scientific explorations, salvages, submarine rescues, and the oil and gas industries. In [1,2,3], the researchers utilized robotics to collect scientific data for marine geoscience about submarine volcanism and hydrothermal vents. Caress et al. employed robotics to detect deep-sea mineral resources and set out the seafloor fiber-optic cables for telecommunications [4]. During the operation of the UUVs, the positioning system is a fundamental unit, which guarantees the efficiency and safety. Recently, with the trend of enhancing UUVs’ automation, and increasing the efficiency of the operation, the positioning systems are expected to perform with greater accuracy, which means that the expected error is less than 0.1 m, with a high update rate, which is faster than 20 Hz. Unfortunately, the conventional positioning solutions hardly meet the mentioned requirements at a modest cost.

It is known that the electromagnetic waves attenuate significantly in the water, which leads a limitation of the communication distance, so the Global Navigation Satellite Systems (GNSSs) [5] and other wireless technologies, such as the Wi-Fi-based Positioning Systems (WPSs) [6], the Bluetooth Positioning Systems (BPSs) [7], and the ultra-wideband (UWB) positioning systems, are not suitable to achieve the underwater localization. Hence, the conventional underwater localization methods are mainly dependent on inertial sensors, DVLs [8], and acoustic devices which have good propagation characteristics in water. As is well known, the inertial sensors mainly refer to the Inertial Measurement Units (IMUs), and are adopted to estimate the displacements and altitudes of UUVs by integrating accelerations and angular speeds over time. Nevertheless, the inertial sensors suffer from unbound drift error, and need to be calibrated beforehand. As for the DVLs, they were developed based on the Doppler effects of the acoustic waves to calculate the speed of the vehicle relative to the seabed by measuring the frequency variation between the emitted wave and the reflected wave [9]. Although DVLs are widely applied in underwater localization implementations, its performance is proportional to the price in general. For example, a modest one costing about USD6k can obtain about 5% accuracy, and the ones with 0.1% accuracy cost around USD20k [10]. Besides that, there is a degradation in the accuracy of DVLs when they are operated in the complex terrain. The acoustic devices mainly include the Long Baseline (LBL) [11], the Short Baseline (SBL) and Ultra-Short Baseline (USBL) positioning systems. However, the implementation of acoustic equipment at close distances might be problematic, since the acoustic signal that bounced off the objects would be detected multiple times by the receiver. In addition to the high cost of its deployment, the slow refresh rate and low resolution are limitations of the acoustic devices.

In addition to adopting acoustic devices for underwater positioning, visual sensors have been used for underwater localization. There are two common visual-based positioning methods; one is the Visual Odometries (VOs) and another is the Visual Simultaneous Localization and Mappings (V-SLAMs). The former is used to establish the motion estimation, the latter can simultaneously build the surrounding maps and achieve the localization. However, pure visual methods are thought to be unreliable in underwater environments, because the features’ extraction and tracking are corrupted by the uncertain illumination conditions and the scattering effect. In addition to that, the amount of features in the subsea is not as much as that on the land. These factors raise challenges in the underwater visual-positioning methods. On the other hand, for the pure monocular camera methods, it is impossible to estimate the trajectory in an absolute scale. For the stereo camera methods, the low precision of the features matching in water prevents the depth maps from being produced by the parallax calculation algorithm, and their detection range is limited by the baseline.

In order to solve these problems, in the paper, a novel underwater positioning approach is proposed. It is developed based on a LiDAR camera and an IMU sensor. More specifically, a monocular LiDAR camera is used to simultaneously provide the RGB images and depth maps. The ORB descriptor is used to detect the features from the RGB image and track them. The depth of an arbitrary pixel in the image plane can be obtained by the depth map. Based on that, the 3D positions of the features can be calculated. Meanwhile, the IMU sensor measures the accelerations and orientations of the vehicle. The information from the LiDAR camera and the IMU sensor is fused to calculate the orientation and trajectory of the UUVs. The experiment results show that the positioning accuracy of the method is less than 0.5 m. There are two main contributions in the paper:

- The first contribution is to construct the underwater measurement model of the LiDAR scanner;
- The second contribution is that a novel underwater visual positioning system based on the LiDAR camera is investigated.

The remainder of the paper is organized as follows. Section 2 introduces the related work in the underwater positioning field. Section 3 shows the experimental platform. Section 4 shows the details of the proposed methodology, and presents some necessary formula derivations. Section 5 explains the implementation of this method, the experimental results are illustrated and the related discussions are described. In Section 6, the conclusions and the future work are presented.

## 2. Related Work in Underwater Visual Positioning

The visual underwater positioning systems, based on monocular and stereo cameras, have been studied extensively. In [12], an underwater self-localization approach based on pseudo-3D vision-inertia was reported. The developed method merged depth information with 2D optical images, and utilized a tightly-coupled nonlinear optimization algorithm to fuse the IMU data and the RGB images from the downward-looking camera. The experimental results illustrated that the proposed method can achieve continuous and robust localization under the changing underwater environment. He et al. investigated a novel underwater navigation method [13], which was based on particle swarm optimization (PSO) and the unscented FastSLAM algorithm. In the method, the PSO was adopted to solve the particles’ degeneracy. The experiment’s results revealed that the method had a better performance compared with FastSLAM in underwater environments. Dabove et al. presented a low-cost monocular visual odometry algorithm for underwater positioning [14]; the simulation experiments showed that this method could acquire effective and reliable results. An improved underwater SLAM algorithm, based on ORB features, was proposed by Lin et al. [15], and the nonlinear optimization method was utilized to optimize the scale of visual odometry and the AUV pose. Xu et al. combined inertial sensors, sonar and a monocular camera to predict the pose and the trajectory of the target [16]. The tank tests proved the approach was effective and reliable. In [17], a naive Convolutional Neural Network (CNN) model was constructed, to learn the global descriptors in images. The designed network can reduce the quantity of the data involved in the process of the global localization and loop closure detection. Through experiments, the method outperforms other approaches in underwater environments. However, the visual positioning systems with the monocular camera are generally considered to estimate motions on a relative scale.

In order to provide the predicted poses and trajectories on an absolute scale, Carrasco et al. investigated a trivial but fast graph-SLAM approach, using a stereo camera [18]. The experiments showed the localization method is effective and suffers from the limited error. Fanfani et al. reported a stereo visual odometry system for underwater positioning [19], using the reliable keypoints and keyframes to achieve an accurate trajectory. A stereo-vision SLAM algorithm for localization, based on point and line features, was developed by Wang et al. [20]; it is suitable to work in feature-poor underwater environments. The method was verified by means of practical experiments. Appelt et al. [21] exploited the Intel T265 stereo camera to realize the visual localization and underwater tracking. Due to the scattering phenomena and the sparse-feature context in water, it is hard for stereo camera-based methods to estimate the depth of features accurately.

Although there are some visual positioning systems using the artificial makers [22,23], the markers are required to be set out beforehand.

Meanwhile, many of the LiDAR SLAM frameworks were reported for ground robotics and drones. The conventional LiDAR SLAM, using the Iterative Closet Point (ICP) method, requires high computational resources and works at a low update rate [24,25]. To overcome the shortcomings, the researchers started to exploit high-efficiency feature extraction methods. The LOAM [26] has drawn much attention, where matching on the edge features and planar features are used to solve the LiDAR SLAM problem for mobile robots. Based on LOAM, the Fast LOAM [27] framework was investigated, which is optimized to cut the computational cost by a third [28]. Simultaneously, Shan et al. proposed a lightweight and real-time pose estimation method, named LeGO-LOAM [29]. The method is able to remove noise using point cloud segment, and intends to achieve the LiDAR SLAM for Unmanned Ground Vehicles (UGVs). The surrounding points from the current frame are segmented into the ground points and non-ground points. Through taking segmentation and two-stage matching, the number of the selected features is reduced by 40% [30]. The experimental results showed that LeGO-LOAM is able to run on a small computing platform at real-time performance. In addition to that, Li et al. proposed the LiLi-OM [31], in which the weighted feature selection method is applied. Lin and Zhang et al. reported a new SLAM, using the LiDAR sensor with a small field of vision [32]. In spite of the many existing well-designed LiDAR SLAM frameworks, the LiDAR techniques are rarely implemented in water.

To summarize the above, the visual positioning systems with only a monocular camera can estimate motions on a relative scale. For stereo camera-based methods, it is hard to accurately estimate the depth of the features, because of the scattering phenomena and the sparse-feature context in water. The methods based on artificial markers require the prior arrangement for the markers. Most of the LiDAR frameworks are developed for ground robots and drones, which are unsuitable for working in water.

In the paper, a visual positioning system is investigated for underwater environments by using the LiDAR camera RealSense L515 and the inertial sensor. Unlike the above frameworks, in the proposed method, the features are detected from the RGB images through the ORB descriptor. The depths of the features are extracted from the aligned depth images generated by the LiDAR scanner. Subsequently, the IMU measurements are fused to estimate motions. In this case, the method is expected to run at low computational complexity with high accuracy and robustness.

## 3. System Overview

**Figure 1:**
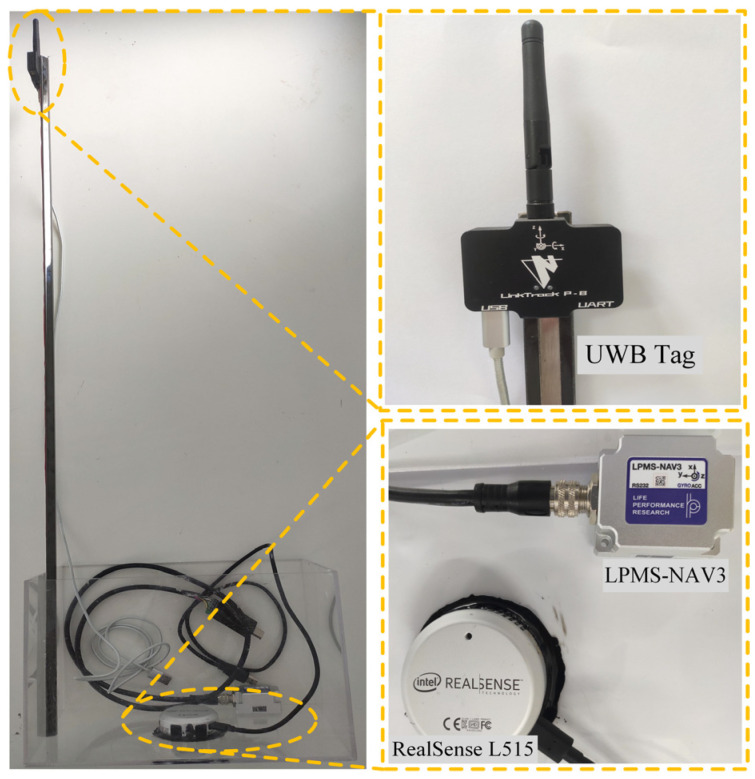
The Layout of Sensors.

**Figure 2:**
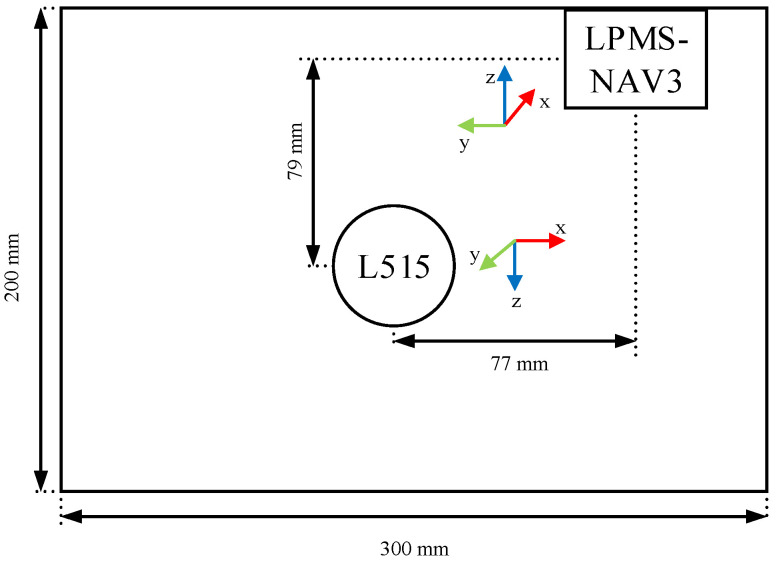
2D position of devices and their coordinates.

The data are collected with the equipment shown in Figure 1. The equipment includes the LPMS-NAV3 IMU sensor, the RealSense L515 LiDAR camera, and the LinkTrack ultra-wideband (UWB) positioning system. The IMU sensor is mainly used to measure the orientation, and the LiDAR camera is applied to capture the colorful images and depth maps of the surrounding environments. The UWB positioning system can give the reference trajectory. The acrylic box is utilized to simulate the UUV to carry the sensors. The LiDAR camera and LPMS-NAV3 are fixed on the bottom of the box, as shown in Figure 1 and Figure 2. The sensors are introduced in detail as follows.

### 3.1. LPMS-NAV3

LPMS-NAV3 is an IMU sensor based on the MEMS technology with a three-axis gyroscope and three-axis accelerometer to measure its rotation rate and acceleration with respect to the inertial frame at 100 Hz. In the paper, the white noise and a slowly varying sensor bias are considered. The rotation matrices are calculated by using Forster et al.’s on-manifold pre-integration theory [33]. Figure 1 shows the layout of the sensors.

### 3.2. Intel RealSense L515

Intel RealSense L515 is a solid-state Light Detection and Ranging (LiDAR) camera with an infrared (IR) laser, a MEMS, and an IR photodiode. It could output the RGB images and depth maps at the same time. The depth point represents the distance of a specific point in the scene from the camera. According to its manual, the depth capture ranges from 0.25 m to 9 m. In addition, its rate of color stream and depth stream frame is up to 30 FPS, which is enough to meet the needs of general UUVs’ applications.

### 3.3. LinkTrack

**Figure 3:**
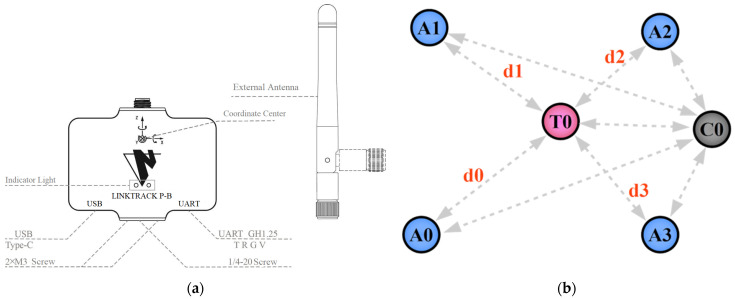
(**a**) Structural diagram of anchor; (**b**) Local positioning working mechanism. The A0 to A3 represent four anchors, T0 is a tag, and C0 denotes a console. The d0 to d3 represent the distance between two nodes.

LinkTrack is an ultra-wideband (UWB) positioning system with good anti-interference ability and stability, and it could provide centimeter-level real-time positioning accuracy in a 3D-positioning environment. It consists of several nodes with its unique ID. In order to obtain a satisfactory performance, the four nodes are set as anchors, which means their locations are fixed. The other one node, named tag, is mounted on the data-collecting device, as shown in Figure 1. The last one works as the console to gather the positioning information of the tag. The layout of the nodes is shown in Figure 3b. Its positioning error is less than 10 cm and the frequency can be up to 200 Hz.

## 4. Methodology

**Figure 4:**
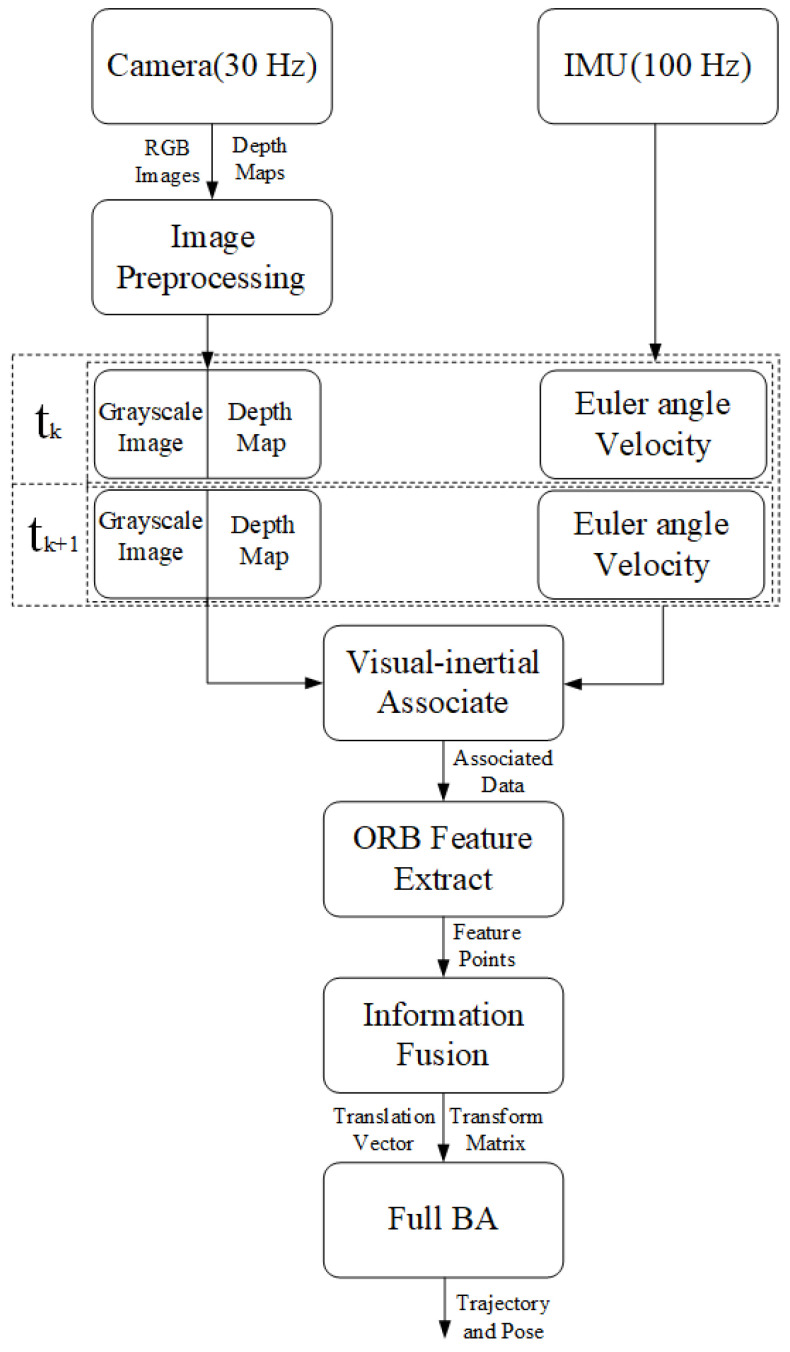
Architecture of the algorithm.

The proposed algorithm architecture is shown in Figure 4. In this approach, the camera images, including RGB images and depth maps, are collected with the live timestamp. After the process of image filtering and grayscale conversion, the quality of the images is improved. The recorded data, including the rotation rate, the RGB images, and the depth maps, are associated by the timestamp. Then, the features are detected and tracked through the ORB descriptor, and are converted into 3D space by using the depth maps. The translation vector between the two continuous frames in a series of RGB images can be calculated. Subsequently, the translation vectors and rotation matrices are corrected by the bundle adjustment algorithm.

**Figure 5:**
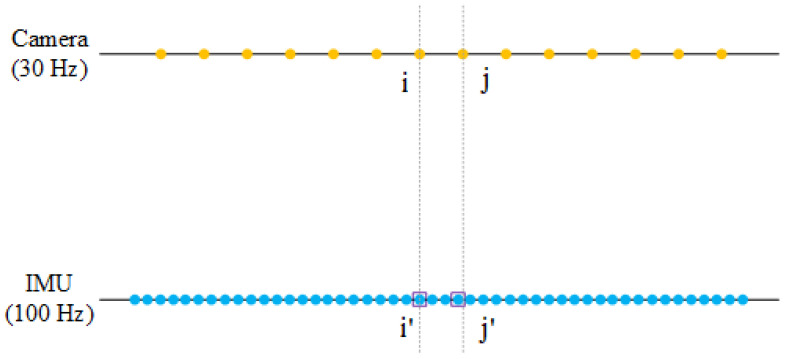
Camera and IMU timestamp association.

In the data association process, the frequency of recording RGB images and depth maps is 30 Hz, while the frequency of the IMU is 100 Hz. In this case, the camera data are intended to be associated with the IMU data by the timestamp. More specifically, as shown in Figure 5, assuming the *i*th camera frame as the starting point, the IMU data *i’* are found by the matching nearest timestamp. A similar process can be completed for the *j*th camera frame. By this means, each frame captured by the camera is expected to find good matches in the IMU data.

As is well known, the camera motion is able to be described by a rotation matrix and a translation vector. The notations and frame definitions used in the paper are: ${\left(\cdot \right)}^{w}$ is denoted as world frame; ${\left(\cdot \right)}^{b}$ represents body frame; ${\left(\cdot \right)}^{c}$ is camera frame; ${\left(\cdot \right)}_{c}^{b}$ defined as transforming from the camera frame to the body frame; ${\left(\cdot \right)}_{w}^{c}$ defined as transforming from the world frame to the camera frame; ${\left(\cdot \right)}^{c_{k}}$ is the *k*th camera frame. In the paper, the IMU frame is identical to the body frame, so it is denoted by same symbol, i.e., ${\left(\cdot \right)}^{b}$. The symbols T, R, and t indicate the transform matrix, rotation matrix, and translation vector respectively, and T∈SE(3), R∈SO(3)  and $t\in {\mathbb{R}}^{3}$.

### 4.1. Underwater Calibration and Depth Correction

The RealSense L515 LiDAR camera was originally designed for implementation in the air. The situation will be different when it is put into the water. Thus, in order to obtain the accurate 3D positions of the features extracted by the ORB descriptor in the RGB images, the underwater calibration and depth correction are essential. While the RealSense camera came with the intrinsic parameters when leaving the factory, its calibration process is completed in the air rather than in the water. In the research, the camera was calibrated in the water by a camera calibration algorithm [34], so that the focal lengths, principal points, and distortion coefficients were accessed properly.

According to the principle of camera imaging, the perspective projection model can be noted as follows:

(1) $$ZP_{uv}=Z\left[\begin{array}{c} u\\ v\\ 1 \end{array}\right]=K\left(RP_{w}+t\right)=K\left(R|t\right)P_{w}^{\prime }=KT^{\prime }P_{w}^{\prime }\;\mathrm{with}\;K=\left[\begin{array}{ccc} f_{x} & 0 & c_{x}\\ 0 & f_{y} & c_{y}\\ 0 & 0 & 1 \end{array}\right],\;P_{w}=\left[\begin{array}{c} X_{w}\\ Y_{w}\\ Z_{w} \end{array}\right],\;P_{w}^{\prime }=\left[\begin{array}{c} X_{w}\\ \begin{array}{c} Y_{w}\\ Z_{w}\\ 1 \end{array} \end{array}\right]$$

where *K* and $T^{\prime }$ are the intrinsic matrix and extrinsic matrix of camera; $P_{w}$ is the position of 3D point in world frame; *Z* represents the depth of 3D point; $P_{uv}$ is the projection of $P_{w}$ in the pixel plane; $f_{x}$ and $f_{y}$ are the focal lengths of *x* and *y* direction; $c_{x}$ and $c_{y}$ are the principal points of the camera.

**Figure 6:**
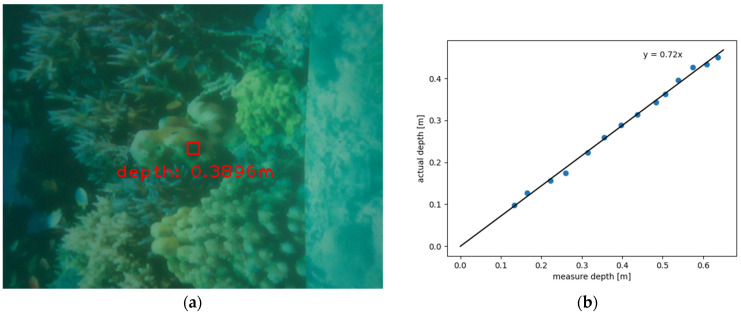
(**a**) Underwater depth test; (**b**) The mathematical relationship of camera underwater ranging.

The parameter *Z* mentioned above could be obtained from the depth map generated by the RealSense L515. The depth measurement is achieved by the laser scanning. In the air, the depth measurement is relatively accurate. However, the speed of light propagation through the water is varied, and the measurement range is also limited, resulting from the underwater scattering phenomenon. To consider the error of the depth measurement in water, a test, as shown in Figure 6a, was conducted. Figure 6b depicts the results, where there is a linear relation between the measure depth and actual depth. By the least square method, as presented in Equations (2) and (3), the linear regression model can be derived. The coefficient b is about 0.72, and the bias coefficient a can be regarded as 0:

(2) $$\bar{y}=bx+a$$

(3) $$b=\frac{\sum_{i=1}^{n}\left(x_{i}-\bar{x}\right)\left(y_{i}-\bar{y}\right)}{\sum_{i=1}^{n}{\left(x_{i}-\bar{x}\right)}^{2}},\;and\;a=\bar{y}-b\bar{x}$$

Compared with the underwater visual positioning methods based on the monocular or stereo camera, in this method, the depth of the arbitrary pixel can be obtained directly by the LiDAR scanner. Since the depth calculation process, which is regarded as being a step of high computational cost, can be skipped, the computational complexity of the proposed method is lower than that of others.

### 4.2. Translation Vector between Dual Frames

As mentioned before, the rotation data measured by the IMU sensor are the orientation of the body frame relative to the inertial frame, which must be transformed to the camera frame. Hence the transformation matrix from the body frame (also the IMU frame) $T_{b}^{c}$ to the camera frame is built as:

(4) $$T_{b}^{c}=\left[\begin{array}{cc} R_{b}^{c} & t_{b}^{c}\\ 0_{1\times 3} & 1 \end{array}\right]\in {\mathbb{R}}^{4\times 4}$$

As shown in Figure 2, the rotation matrix $R_{b}^{c}$ is accessible, and it can be acquired from the Euler angle ${\left[\begin{array}{ccc} \frac{\pi }{2} & 0 & \pi \end{array}\right]}^{\;}$ in *ZYX* order. The translation vector $t_{b}^{c}$ can be denoted as ${\left[\begin{array}{ccc} -0.077 & 0.079 & 0 \end{array}\right]}^{T}$ (unit: meter) by manual measurement.

So far, the RGB and depth images are obtained from the camera, and the rotation information is acquired from the IMU. Through Equation (5), the relative poses of the camera and the IMU could be attained:

(5) $$T_{w}^{c}=T_{b}^{c}\cdot T_{w}^{b}=T_{b}^{c}\cdot {\left(T_{b}^{w}\right)}^{-1}$$

(6) $$T_{c_{k}}^{c_{k+1}}=T_{w}^{c_{k+1}}\cdot T_{c_{k}}^{w}=T_{w}^{c_{k+1}}\cdot {\left(T_{w}^{c_{k}}\right)}^{-1}\;\mathrm{with}\;T_{c_{k}}^{c_{k+1}}=\left[\begin{array}{cc} R_{c_{k}}^{c_{k+1}} & t_{c_{k}}^{c_{k+1}}\\ 0_{1\times 3} & 1 \end{array}\right]\in {\mathbb{R}}^{4\times 4}$$

The symbol $T_{c_{k}}^{c_{k+1}}$ is the transform matrix between *k*th and (*k* + 1)th camera frame. The rotation matrix $R_{c_{k}}^{c_{k+1}}$ is the rotation matrix between dual frames. The $t_{c_{k}}^{c_{k+1}}$ is the translation vector between two frames, which is calculated by the multiple-view geometric techniques. The Perspective-n-Point (PnP) algorithm and the camera projection model are employed to acquire the feature points’ actual position in the camera frame. The n reliable feature points are selected, which are denoted as $P_{i}^{c_{k}}$ and $P_{i}^{c_{k+1}}$. The $R_{c_{k}}^{c_{k+1}}$ is adopted to solve the translation vector $t_{c_{k}}^{c_{k+1}}$ between the dual frames, and the average $t_{c_{k}}^{c_{k+1}}$ describes the corresponding translation vector. Hence, by iterating the transform matrix, the complete trajectory of facility is available. The equations are given as follows:

(7) $$t_{c_{k}}^{c_{k+1}}=\frac{1}{n}\sum_{i=1}^{n}\left(P_{i}^{c_{k+1}}-R_{c_{k}}^{c_{k+1}}P_{i}^{c_{k}}\right)$$

(8) $$T_{c_{1}}^{c_{k+1}}=T_{c_{k}}^{c_{k+1}}\cdot T_{c_{k-1}}^{c_{k}}\cdots T_{c_{2}}^{c_{3}}\cdot T_{c_{1}}^{c_{2}}$$

where the $T_{c_{1}}^{c_{k+1}}$ represents the transform matrix from initial pose to (*k* + 1)th pose of camera; and the *c*_1_ is the initial pose of the camera. Through the iteration of continuous frames, the final pose of the camera, relative to the initial frame, can be acquired.

From the given equations above, the pose of the camera in each of the dual frames can be acquired by the collected images and IMU data; subsequently, the trajectory is accessible. Attributing to the linear transformation, the computational complexity in the step is only *O(n)*, *n* is the number of the features. However, the camera pose in two frames is subjected to the drift error, which accumulates over time. Therefore, an optimization method should be adopted to reduce the error.

### 4.3. Bundle Adjustment

To acquire a more accurate translation vector and rotation matrix of the camera, as well as the feature points position, the bundle adjustment algorithm was utilized to achieve this target [35]. Assuming that there are n 3D points $P_{j}={\left[\begin{array}{ccc} X_{j} & Y_{j} & Z_{j} \end{array}\right]}^{T}$, and their projection points $p_{j}={\left[\begin{array}{cc} u_{j} & \begin{array}{cc} v_{j} & 1 \end{array} \end{array}\right]}^{T}$ in the pixel coordinates, $p_{i}$ is given by Equation (1). Moreover, because of being influenced by the unknown camera pose and the observation noise, the reprojection error in Equation (10) is:

(9) $$z_{ij}\overset{\text{def}}{=}{\left[\begin{array}{cc} u_{i} & v_{i} \end{array}\right]}^{T}$$

(10) $$e_{ij}=z_{ij}-h\left(T_{i},\;P_{j}\right)$$

where $z_{ij}$ is the *j*th observed point in the *i*th pose; h(·) denoted as projection from 3D point to imaging plane; $T_{i}$ is the *i*th pose of camera; and $p_{j}$ is the *j*th point; and $e_{ij}$ represents the reprojection error.

The main target of the bundle adjustment is minimum reprojection errors, as presented in Equation (11). Generally, when minimizing the reprojection error, the camera poses and points position are the variables for optimization, so that a partial derivative with respect to camera pose and landmark position should be calculated:

(11) $$T^{*}=argmin\frac{1}{2}\sum_{i=1}^{m}\sum_{j=1}^{n}\|e_{ij}{\|}^{2}=\frac{1}{2}\sum_{i=1}^{m}\sum_{j=1}^{n}\|z_{ij}-h{\left(T_{i},P_{j}\right)\|}^{2}$$

(12) $$\frac{1}{2}\|f{\left(x+∆x\right)\|}^{2}\approx \frac{1}{2}\sum_{i=1}^{m}\sum_{j=1}^{n}\|e_{ij}+F_{ij}∆\xi_{i}+E_{ij}∆P_{j}\|{}^{2}$$

where $F_{ij}$ and $E_{ij}$ are the partial derivative of camera pose and feature point, with respect to cost function. The derivation is as follows:

(13) $$P_{j}^{\prime }={\left(TP_{j}\right)}_{1:3}=RP_{j}+t={\left[\begin{array}{ccc} X_{j}^{\prime } & Y_{j}^{\prime } & Z_{j}^{\prime } \end{array}\right]}^{T}$$

(14) $$F_{ij}=\frac{\partial e_{ij}}{\partial \delta \xi_{i}}=\underset{\delta \xi_{i}\to 0}{\lim}\frac{e_{ij}\left(\delta \xi_{i}⨁\xi_{i}\right)-e_{ij}\left(\xi_{i}\right)}{\delta \xi_{i}}=\frac{\partial e_{ij}}{\partial P_{j}^{\prime }}\frac{\partial P_{j}^{\prime }}{\partial \delta \xi_{i}}$$

(15) $$\frac{\partial e_{ij}}{\partial P_{j}^{\prime }}=-\left[\begin{array}{ccc} \frac{\partial u}{\partial X_{j}^{\prime }} & \frac{\partial u}{\partial Y_{j}^{\prime }} & \frac{\partial u}{\partial Z_{j}^{\prime }}\\ \frac{\partial v}{\partial X_{j}^{\prime }} & \frac{\partial v}{\partial Y_{j}^{\prime }} & \frac{\partial v}{\partial Z_{j}^{\prime }} \end{array}\right]=-\left[\begin{array}{ccc} \frac{f_{x}}{Z_{j}^{\prime }} & 0 & -\frac{f_{x}X_{j}^{\prime }}{Z_{j}^{\prime 2}}\\ 0 & \frac{f_{y}}{Z_{j}^{\prime }} & -\frac{f_{y}Y_{j}^{\prime }}{Z_{j}^{\prime 2}} \end{array}\right]\in R^{2\times 3}$$

(16) $$\frac{\partial P_{j}^{\;}}{\partial \delta \xi_{i}}={\left(TP_{j}\right)}^{⨀}=\left[\begin{array}{cc} I & -P_{j}^{\prime ⋀}\\ 0^{T} & 0^{T} \end{array}\right]\in {\mathbb{R}}^{4\times 6},\;\frac{\partial P_{j}^{\prime }}{\partial \delta \xi_{i}}=\left[\begin{array}{cc} I & -P_{j}^{\prime ⋀} \end{array}\right]\in {\mathbb{R}}^{3\times 6}$$

(17) $$\frac{\partial P_{j}^{\;}}{\partial \delta \xi_{i}}=-\left[\begin{array}{ccccc} \frac{f_{x}}{Z_{j}^{\prime }} & 0 & -\frac{f_{x}X_{j}^{\prime }}{Z_{j}^{\prime 2}}-\frac{f_{x}X_{j}^{\prime }Y_{j}^{\prime }}{Z_{j}^{\prime 2}} & f_{x}+\frac{f_{x}X_{j}^{\prime 2}}{Z_{j}^{\prime 2}} & -\frac{f_{x}Y_{j}^{\prime }}{Z_{j}^{\prime }}\\ 0 & \frac{f_{y}}{Z_{j}^{\prime }} & -\frac{f_{y}Y_{j}^{\prime }}{Z_{j}^{\prime 2}}-f_{y}-\frac{f_{y}Y_{j}^{\prime 2}}{Z_{j}^{\prime 2}} & \frac{f_{y}X_{j}^{\prime }Y_{j}^{\prime }}{Z_{j}^{\prime 2}} & -\frac{f_{y}X_{j}^{\prime }}{Z_{j}^{\prime }} \end{array}\right]\in R^{2\times 6}$$

(18) $$E_{ij}=\frac{\partial e_{ij}}{\partial P_{j}}=\frac{\partial e_{ij}}{\partial P_{j}^{\prime }}\frac{\partial P_{j}^{\prime }}{\partial P_{j}}=-\left[\begin{array}{ccc} \frac{f_{x}}{Z_{j}^{\prime }} & 0 & -\frac{f_{x}X_{j}^{\prime }}{Z_{j}^{\prime 2}}\\ 0 & \frac{f_{y}}{Z_{j}^{\prime }} & -\frac{f_{y}Y_{j}^{\prime }}{Z_{j}^{\prime 2}} \end{array}\right]R\in {\mathbb{R}}^{2\times 3}$$

where ${\left(\cdot \right)}_{1:3}$ means the submatrix with the first row to the third row; ⨁ is left multiplication perturbations on Lie algebras; ⨀ represents the transformation of a point in homogeneous space into a 4×6 matrix.

The Gauss–Newton method is adopted, to solve the least squares problem. The reprojection error can be minimized, as follows:

(19) $$H∆x=g\;\mathrm{and}\;H=J_{ij}^{T}\cdot J_{ij}\;\mathrm{with}\;J_{ij}=\left[\begin{array}{cc} F_{ij} & E_{ij} \end{array}\right]=\;\left(\begin{array}{cccccccccc} 0_{2\times 6} & \cdots & 0_{2\times 6} & \frac{\partial e_{ij}}{\partial \delta \xi_{i}} & 0_{2\times 6}\;\cdots & 0_{2\times 3} & \cdots \;0_{2\times 3} & \frac{\partial e_{ij}}{\partial P_{j}} & \cdots & 0_{2\times 3} \end{array}\right)$$

It is not hard to prove that H is a sparse matrix; by utilizing the sparseness and marginalization approaches [36], the appropriate camera poses and feature points position could be derived.

### 4.4. Error Analysis

In practical situations, the proposed iterative method is subjected to the accumulated error, which indicates that the drift error between two continuous frames is accumulated as time goes by. The drift error is mainly caused by electronic noise from the camera and the IMU sensor. The camera also produces the noise in the film grain and in the unavoidable shot noise of an ideal photon detector. There are other factors giving rise to the error between two continuous frames, including lens distortion, features mismatching, and deviations in depth measurement. The lens distortion is caused, especially in wide-angle lenses, by trying to capture more of the view in the image. Although the camera calibration procedure can reduce the effects of distortion, the effects cannot be erased totally. As for the features mismatching problem, the current algorithms cannot guarantee that all of the features are tracked correctly. In the depth measurement process, the electronic noise and the scattering phenomenon for the scanning laser both attribute to the deviation, because the accumulated error is monotone increasing and unbounded. In the method, the BA method is utilized to reduce the drift error, which can slow down the rising rate of the accumulated error.

## 5. Experiments and Discussion

In the implementation of the method, the codes were written in C++. The section of image processing, feature detection, and tracking, was developed based on the OpenCV libraries. The two threads were carried out to accelerate the whole process. The main thread was utilized to estimate the trajectory by calculating the translation vector and processing the BA algorithm. The second thread was employed to display the predicted trajectory and the UUVs’ orientation.

**Figure 7:**
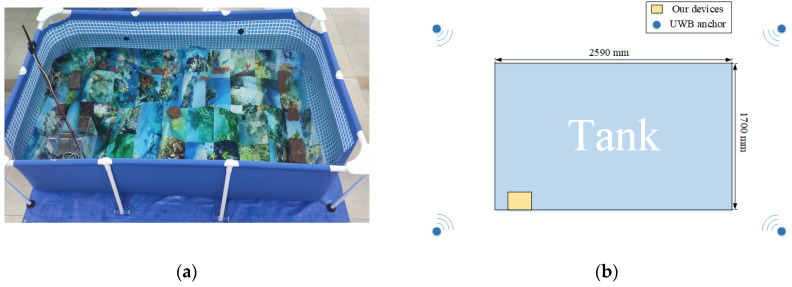
(**a**) The experiment environment; (**b**) The layout of experiment environment.

To verify that our algorithm was effective and available in an underwater environment, a series of experiments were conducted in the tank. The size of the experimental tank was 2590 mm × 1700 mm × 610 mm shown in Figure 7a. At the bottom of the tank, the posters were laid to simulate the actual seabed environment with artificial starfishes and sea weed. Besides that, some of the objects, such as starfishes, seaweeds, and artificial structures were also placed on the bottom. In this case, the experimental tank can be considered to be analogous to the real underwater environments. The data collection equipment was introduced in Section 3. The arrangement of the UWB system is shown in Figure 7b: the four UWB positioning anchors are fitted around the tank; the UWB positioning tag is mounted on the data recording device. They were all set up over the tank, so that the obstruction for electromagnetic radiation can be avoided.

In the experiments, the data recording device can be regarded as the UUV, and the operator controlled the device manually in the tank to ensure that it traveled along the closed paths. In total, there were four different testing paths, which are shown as rectangular-shaped, triangle-shaped, right-triangle-shaped, and ‘X’-shaped. The proposed method was evaluated twice on each path. During the data collecting, the external UWB positioning system could simultaneously measure the 3D positions of the data recording device. However, as mentioned before, the tag node and camera were in different frames, in order to compare their performances; the original UWB positioning data must be converted to the camera frame by the transformation matrix. Because the arrangement of the UWB positioning system is known, the matrix is derived trivially.

Since the paths which the data collection device travelled along were designed to be closed, the estimated trajectories were expected to be the closed loops. That means that the end points should return to the start points in the results. However, being subject to the accumulation and manual errors, it is inevitable for an offset to exist between the start points and end points. In order to evaluate the experimental results quantitatively, the error ratio is proposed to analyze the performances of the developed method, which is defined as Equation (20):

(20) $$e_{r}=\frac{\mathrm{offset}}{\mathrm{trajectory}\;\mathrm{length}}$$

It is noted that the UWB positioning system can only give the reference trajectories rather than the actual trajectories. In ideal situations, the accuracy of the UWB positioning system is expected to be within 10 cm. However, in the laboratory, where there are many metal racks, its accuracy is degraded. In addition, due to its low updating frequency, the UWB scarcely locates the moving object with high accuracy. The UWB positioning tag, on the top of the stick which is fixed with the data recording device, sometimes moves fast. Hence, when the device is stationary, the measured UWB positioning data is to be trusted more.

**Figure 8:**
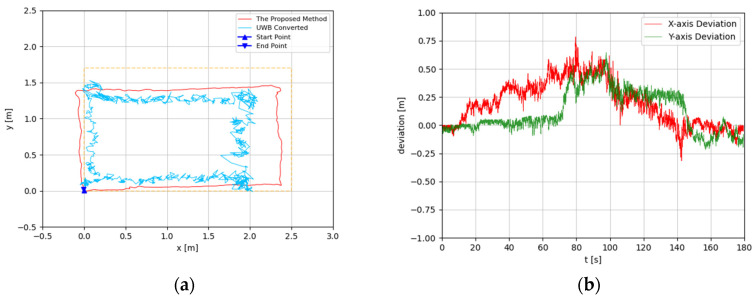
(**a**) Trajectory of rectangle-shaped the first test; (**b**) Positioning comparison of rectangle-shaped the first test.

**Figure 9:**
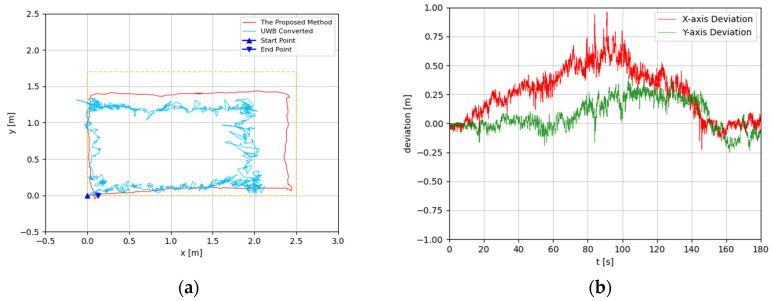
(**a**) Trajectory of rectangle-shaped the second test; (**b**) Positioning comparison of rectangle-shaped the second test.

**Figure 10:**
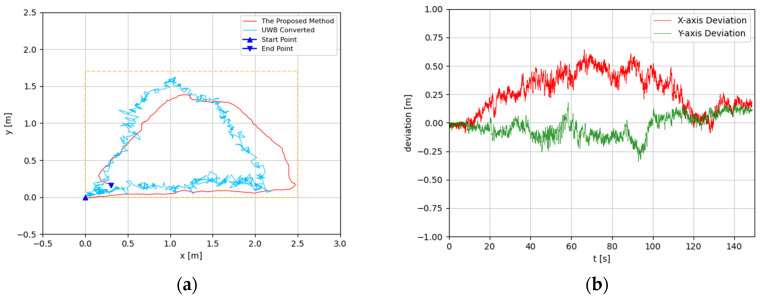
(**a**) Trajectory of triangle-shaped path in the first test; (**b**) Positioning comparison of rectangle-shaped path in the first test.

**Figure 11:**
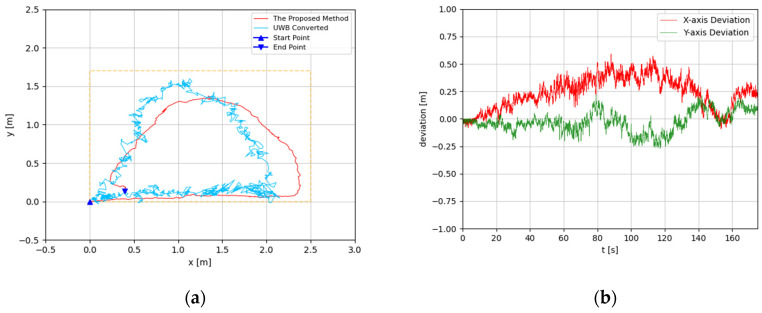
(**a**) Trajectory of triangle-shaped path in the second test; (**b**) Positioning comparison of triangle-shaped path in the second test.

**Figure 12:**
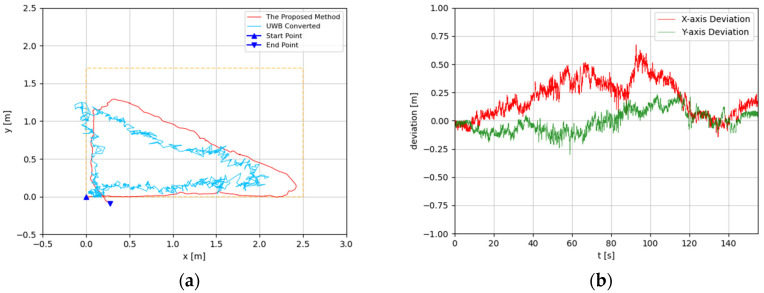
(**a**) Trajectory of right-triangle-shaped path in the first test; (**b**) Positioning comparison of right-triangle-shaped path in the first test.

**Figure 13:**
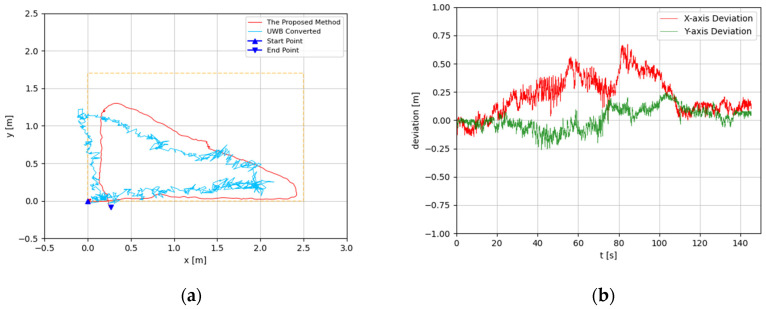
(**a**) Trajectory of right-triangle-shaped path in the second test; (**b**) Positioning comparison of right-triangle-shaped path in the second test.

**Figure 14:**
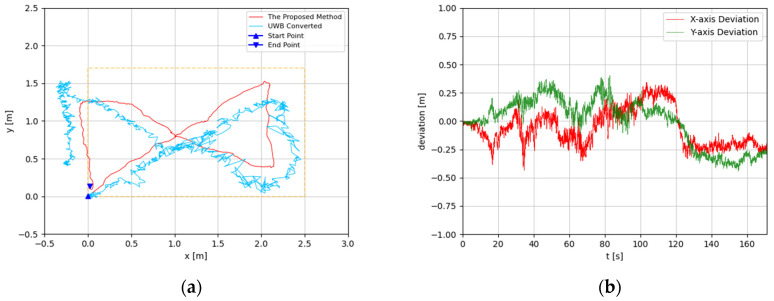
(**a**) Trajectory of ‘X’-shaped path in the first test; (**b**) Positioning comparison of ‘X’-shaped path in the first test.

**Figure 15:**
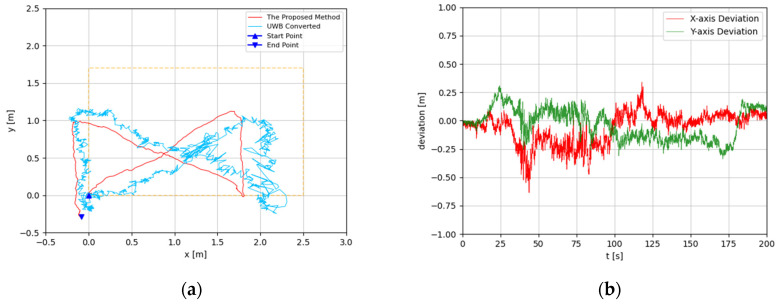
(**a**) Trajectory of ‘X’-shaped path in the second test; (**b**) Positioning comparison of ‘X’-shaped path in the second test.

The trajectories were estimated in 3D space. Since the altitude variation of the recording device was slight, the predicted 3D trajectories were projected to 2D plots. The experimental results are illustrated in Figure 8, Figure 9, Figure 10, Figure 11, Figure 12, Figure 13, Figure 14 and Figure 15. In the Figures related to the trajectories, the orange dot line demonstrates the edges of the experimental tank. In addition, the deviations in the *x*-axis and *y*-axis between the proposed method and the UWB system are presented.

The trajectory length and error ratio of each test are presented in Table 1.

In Figure 8 and Figure 9, the device was operated to travel along the tank, which is a rectangle-shaped path. The estimated trajectory of the proposed method is close to the actual size of the tank, and its start point and end point were nearly coincident; the error ratio was 0.28% and 1.57%, respectively, in the two experiments. With comparison between the proposed method and UWB positioning system, the greatest deviation was about 0.5 m, from 60 s to 120 s. At the end of the experiment, the deviation had decreased to less than 0.1 m.

In Figure 10 and Figure 11, the proposed method is evaluated for the general triangle-shaped path. In Figure 12 and Figure 13, the method is verified for the right-triangle-shaped path. The estimated trajectories draw a triangle shape clearly, and the offset of the start point and end point was about 0.35 m. The error ratio was around 5%. In the comparison between the developed approach and UWB positioning system, the largest deviation was around 0.5 m from 80 s to 100 s. Towards the end of the experiment, the deviation was about 0.1 m.

In Figure 14 and Figure 15, the experiment results of the ‘X’-shaped path are depicted. In this experiment, the device was manually controlled to travel along the diagonal and short edge of the tank. The predicted trajectory of the developed method is close to the desired path in general, and the deviation between start point and end point is about 0.2 m. The error ratio was less than 4.03%. In the positioning comparison between the developed method and UWB positioning system, the greatest deviation was 0.5 m approximately from 35 s to 50 s, while most of the deviations are less than 0.25 m. In the end, the deviations were less than 0.1 m.

According to the results, the estimated trajectories by the proposed method are consistent with the intended paths. The deviations between the proposed method and the UWB system generally increase in the middle segment and decrease at the end. This is because, when the UWB positioning tag is moving, the positioning accuracy is reduced dramatically. At the beginning and the end of each experiment, the device with the UWB positioning tag fixed is static. Hence, at these times, the results of the UWB system are more credible. As discussed before, the proposed method is subjected to accumulated error, which means its drift error will rise over time. Based on that, the error of the proposed method is monotone increasing. Therefore, the deviations between the proposed method and the UWB near the end of the experiment should be considered more seriously. Through the results, the deviations at the end of the experiments are generally less than 0.2 m. In Figure 8b and Figure 15b, the deviations are even less than the 0.1 m. As the accuracy of the UWB positioning system in ideal conditions is about 0.1 m, the results proved that the proposed positioning system is highly accurate. Furthermore, the error ratios were calculated to also evaluate the performances of the proposed methods. The largest error ratios were less than 5%, which indicate the stability and accuracy of the developed method.

## 6. Conclusions

In this paper, a novel positioning system, based on the LiDAR camera for UUVs, was proposed. Through the proposed depth measurement model of the LiDAR camera, the features extracted by the ORB descriptor in RGB images are converted to 3D space. Subsequently, with the orientation from the IMU sensor, the translation vector can be calculated via transformation matrices. The bundle adjustment algorithm is adopted to improve its accuracy by recalculating the rotation matrix and the translation vector. The discrete accuracy and robustness of the method is verified by the practical experiments.

In future works, we would focus on improving the performance of the method in turbid environments, by using an appropriate image enhancement method. Meanwhile the retracking and loop closure detection modules are to be integrated into the method, which can restrict the drift error in dynamic environments.

## Figures and Tables

**Table 1:** Trajectory length and error ratio of each test using the proposed method.

Shape	Trajectory Length (m)	Offset (m)	Error Ratio (%)
Rectangle	7.90	0.02	0.28
	8.18	0.12	1.57
Triangle	6.71	0.34	5.12
	6.77	0.42	6.19
Right-triangle	7.00	0.29	4.11
	7.11	0.28	3.92
‘X’	8.05	0.13	1.67
	7.40	0.29	4.03

## Data Availability

Not applicable.
